# *Carex diaoluoshanica* (*Carex* sect. *Lageniformes*, Cyperaceae), a New Species from Hainan, China

**DOI:** 10.1371/journal.pone.0097658

**Published:** 2014-06-17

**Authors:** Hubiao Yang, Qinglong Wang, Changjun Bai, Xiaoxia Li, Guodao Liu

**Affiliations:** 1 Tropical Crops Genetic Resources Institute, Chinese Academy of Tropic Agricultural Sciences, Danzhou, Hainan, People's Republic of China; 2 Environment and Plant Protection Institute, Chinese Academy of Tropical Agricultural Sciences, Haikou, Hainan, People's Republic of China; National Institute for Viral Disease Control and Prevention, CDC, China, China

## Abstract

*Carex diaoluoshanica*, a new species of *Carex* sect. *Lageniformes* from Hainan, China, is described and illustrated. The new species is similar to *C. breviscapa* but differs in having wider leaves with the leaf base gradually narrowed, 5–10 cm long and petiolelike, culms subfiliform, with only two spikes, the lateral female spikes from near the culm base.

## Introduction

The genus *Carex* was established by Carl Linnaeus *Species Plantarum* (1753). It is one of the largest genera of vascular plants, comprising about 2000 species distributed in various habitats, almost worldwide in distribution[1]–[4]. In China, it is represented by 527 species in three subgenera and 69 sections. Nine, additional species have been reported since publication of the Flora of China account[5]–[13].

The genus *Carex* is clearly distinguished from other genera of the Cyperaceae in having consistently unisexual flowers and a perigynium, the latter a sac-like structure of prophyllar origin that surrounds the naked gynoecium [14]. The variations in the structure of the perigynium are used as the key features in *Carex*. This is largely due to the subtle differences in shape, size, texture and nervation, which have been used as primary characters for the delimitation of many species in *Carex* [15]. *Carex* has been divided into subgenera in a number of ways based on the characters of stigma number, inflorescence structure and the distribution of staminate and pistillate flowers within the spikes. The most influential was Georg Kükenthal's classification which recognised four subgenera: *Carex* subg. *Car*ex, *C*. subg. *Indocarex*, *C*. subg. *Vignea* and *C*. subg. *Primocarex*. Subsequently, *C*. subg. *Indocarex* and *C*. subg. *Primocarex* were corrected to *C*. subg. *Vigneastra* [16]. This classification was widely followed by most authors[3], [8], [17], [18], [22], [23].

*Carex* sect. *Lageniformes* (Ohwi) Nelmes belongs the core *Carex* clade[23]. It is characterized by having leaves longer than the culm, the terminal spike male and lateral spikes female, the female glumes oblong-ovate or obovate, the perigynium fusiform or rhombic-fusiform, the nutlet rhombic or fusiform with the apex truncate or shallowly concave, and the style base cylindric, slightly thickened, and persistent. It consists of 12 species mainly distributed in eastern and southeast Asia, with 8 species reported in China, 4 of which are endemic [3], [8]. Hainan Island is located at the southern part of China, at the northern edge of tropical Asia, with about 4100 vascular plants species [19]–[20]. To date, 24 species of *Carex* have been reported from Hainan Island [8], [13], [21].

## Materials and Methods

### Ethics statement

The new species reported in this work is collected from Diaoluo Shan Nature Reserve which is protected by the Forestry Bureau of Hainan. Permissions to visit location and field activities were obtained from Nature reserve management.

### Morphological observations

Morphological description of the new species was based on examination of fresh and pressed specimens. Details of the terminal spike staminate, lateral spikes pistillate, pistillate glume, perigynium and nutlet were examined and photographed under a stereomicroscope (Olympus SZX16-6156). The Perigynium and Nutlet shape of *Carex diaoluoshanica*, *C. breviscapa* and *C. longipetiolata* were observed using a Philips XL-30E scanning electron microscope (SEM). The studied specimens are kept in the Herbarium of South China Botanical Garden, the Chinese Academy of Sciences (IBSC), and the Tropical Crops Genetic Resources Institute, Chinese Academy of Tropical Agricultural Sciences (TCGRI).

### Nomenclatural Acts

The electronic version of this article in Portable Document Format (PDF) in a work with an ISSN or ISBN will represent a published work according to the International Code of Nomenclature for algae, fungi, and plants, and hence the new names contained in the electronic publication of a PLOS ONE article are effectively published under that Code from the electronic edition alone, so there is no longer any need to provide printed copies.

In addition, new names contained in this work have been submitted to IPNI, from where they will be made available to the Global Names Index. The IPNI LSIDs can be resolved and the associated information viewed through any standard web browser by appending the LSID contained in this publication to the prefix http://ipni.org/. The online version of this work is archived and available from the following digital repositories: PubMed Central, LOCKSS.

## Results

**Figure 1:**
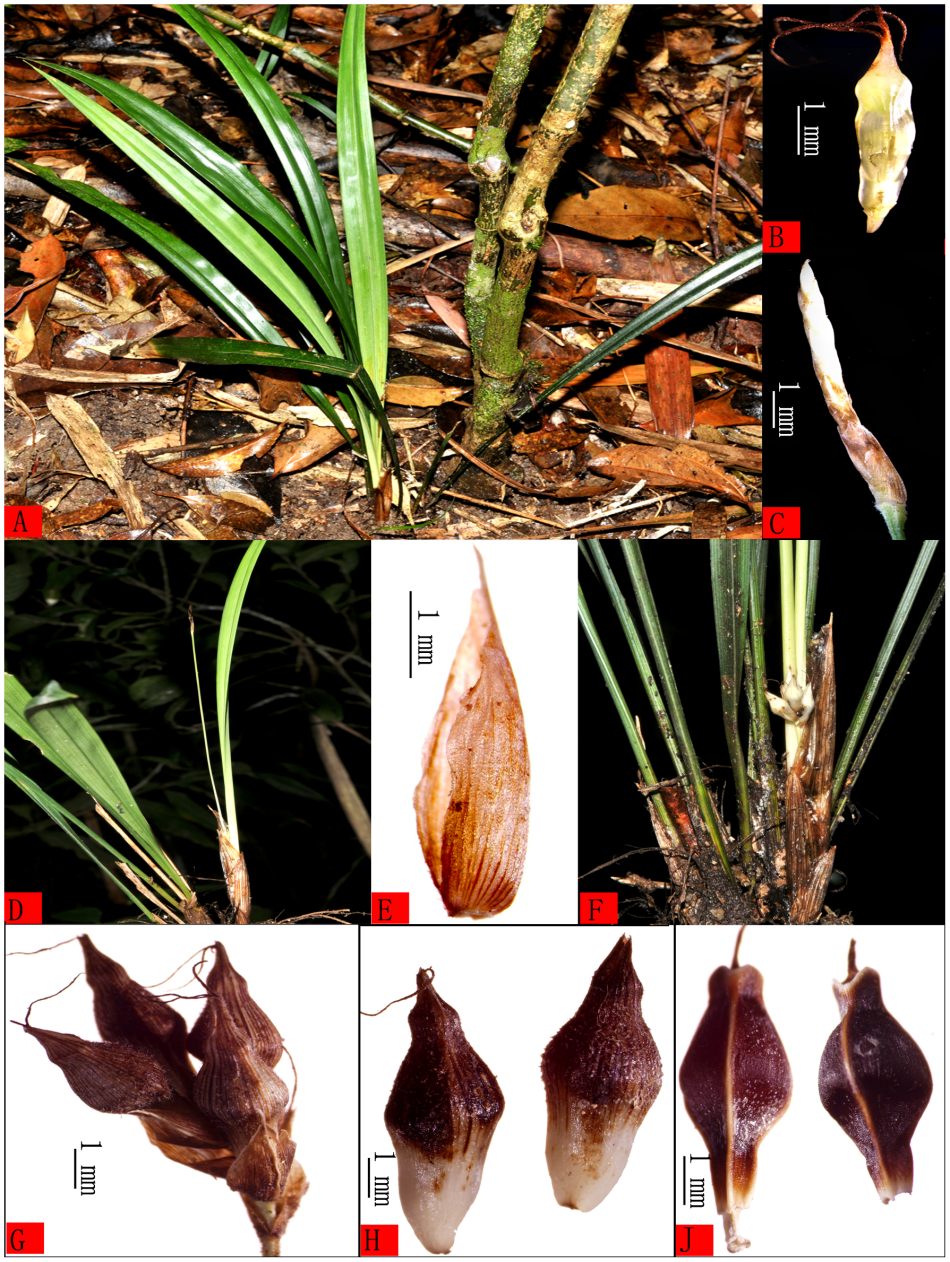
*Carex diaoluoshanica*. A. Habit. B. Stigmas. C. Terminal staminate spike. D. Inflorescence(pistillate spikes enclosed in bladeless sheaths). E. Pistillate glume. F. Flowering phase lateral pistillate spikes. G. Fruit period lateral pistillate spikes. H. Perigynium. J. Nutlet. Photographs by Hu-biao Yang.

During an investigation of the flora of Diaoluo Shan Nature Reserve in 2013, a new species belonging to *Carex* sect. *Lageniformes* was found (Fig. 1). This species is similar to *C. breviscapa* C. B. Clarke, but differs sufficiently to be recognized as a new species. Nine specimens (one holotype, four isotypes and four paratypes) of the studied are kept in the Herbarium of South China Botanical Garden, the Chinese Academy of Sciences (IBSC), and the Tropical Crops Genetic Resources Institute, Chinese Academy of Tropical Agricultural Sciences (TCGRI). Based on the shape of the perigynium and nutlet, the new species is most similar to *Carex breviscapa*, but they differ distinctly as follows (the former is new species, the latter is *C. breviscapa*): culms subfiliform, ca. 0.5 mm thick (culms slightly scabrid, ca. 2–5 mm thick); leaf blades 7–15 mm wide with the base attenuate and petiolelike, ca. 5–10 cm long (leaf blades 6–7 mm wide, not attenuate at base); inflorescence with just 2 spikes (inflorescence 3–5-noded, with 3–5 spikes at each node); lateral spikes 1–1.5 cm long (3–4 cm long), lateral female spike solitary, arising from near culms base and enclosed within bladeless sheaths (lateral female spikes many, arising from well above culm base).

### Taxonomic treatment

**Carex diaoluoshanica H. B. Yang, G. D. Liu & Q. L. Wang sp. nov.**

**Figure 2:**
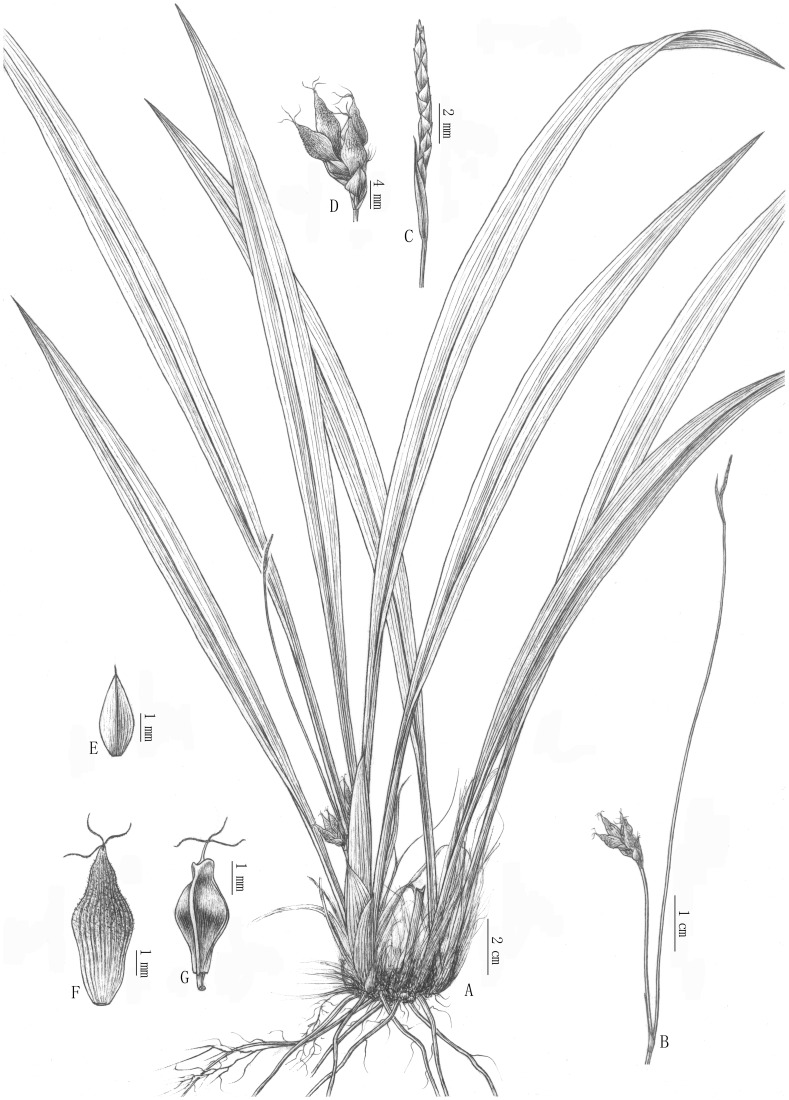
*Carex diaoluoshanica*. A. Hahit; B. Inflorescence; C. Terminal spike staminate; D. Lateral spikes pistillate; E. Pistillate glume; F. Perigynium; G. Nutlet. Drawn by Yu-xi Zhu based on *Yang Hubiao 20130604017* (holotype TCGRI).

**Figure 3:**
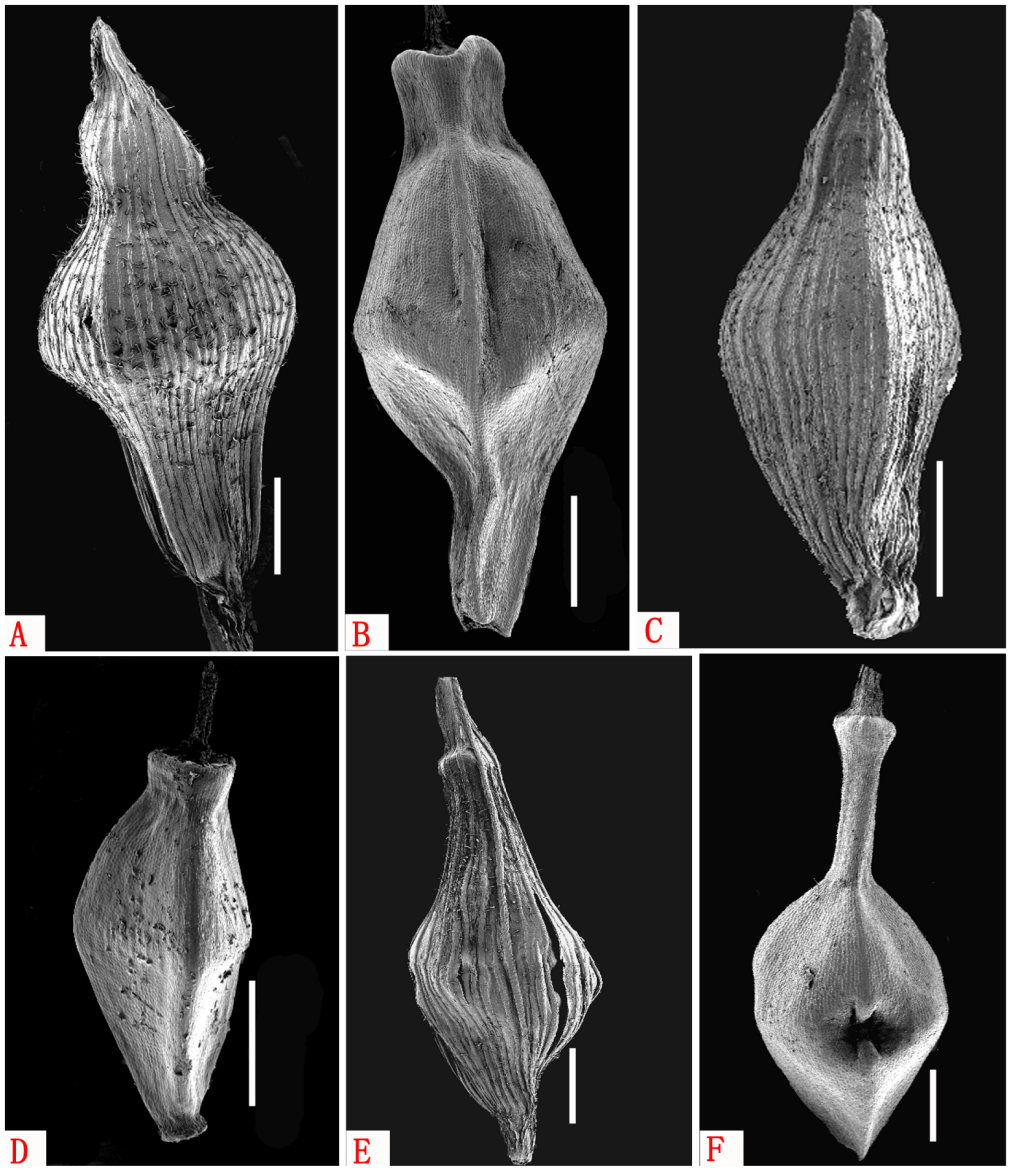
Perigynium and Nutlet shape of *Carex diaoluoshanica*(A and B), *C. breviscapa*(C and D) and *C. longipetiolata*(E and F) viewed under SEM. Scale bars = 1 mm.

**[urn:lsid:ipni.org:names:** 77137990-1**] (** **Figures 1**–**3** **). Type**: —CHINA. Hainan: Lingshui County, Diaoluo Shan Nature Reserve, moist place under forest, alt. 800–900 m, 4 June 2013, *Yang Hubiao 20130604017* (holotype, IBSC; one isotypes, IBSC; three isotypes, TCGRI).

### Description

Perennial; rhizome short, ligneous, covered with dark brown fibrous remains of old leaf sheath. Leaves basal, blade green, ensiform, 10–45×0.7–1.5 cm, flat, glabrous on both surfaces, apex acuminate, gradually narrowed into petiole-like base ca. 5–10 cm long. Culms solitary, arising centrally from leafy shoot, 9–20 cm tall, ca. 0.5 mm thick, subfiliform, trigonous, base clothed with bladeless brown sheaths. Inflorescence with 2 spikes; terminal spike male, narrowly cylindrical, 8–15 mm long, ca. 2 mm thick, sessile; lateral spikes female, peduncle arising from culm base, subfiliform 1–3 cm long, trigonous, enclosed in bladeless brown sheaths, spike cylindric, 10–15 mm long, loosely flowered. Male glumes oblong, ca. 3 mm long, brownish yellow; stamens 3, filaments shorter than the glumes; Female glumes pale yellow, obovate, ca. 4 mm long, membranous, hairless, many-veined, middle vein excurrent into a short awn ca. 0.5–0.8 mm long. Perigynium initially pale yellow, after maturity pale yellow below middle, brown above, longer than the glumes, fusiform, obscurely trigonous, ca. 7 mm long, minutely pubescent, many-veined, base cuneate, stipitate, apex gradually narrowed into a scabrous beak ca. 1–1.5 mm long, orifice straight, 2-toothed; stigmas 3. Nutlet brown to dark brown with angles and stipe yellowish white, tightly enveloped by the perigynium, fusiform, trigonous, 4–5 mm long, base stipitate, stipe 0.4–0.8 mm long, apex with distinct short neck ca. 0.5 mm long, neck obscurely trigonous, shallowly concave at apex; style base slightly thickened, persistent. Fl. and fr. May–July.

### Distribution and Habitat

*Carex diaoluoshanica* is known only from Diaoluo Shan Nature Reserve, Hainan, China. It grows in a thick litter-fall layer with rich organic matter under the tropical mountain rain forest at altitudes of 800–900 m. The population, which comprises approximately 2 500 caespitose individuals, covers an area of 1 000 m^2^. Associates include trees of *Altingia obovata* Merrill & Chun, *Cryptocarya maclurei* Merrill, *Dacrydium pectinatum* de Laubenfels, *D. imbricatus* var. *patulus* de Laubenfels, *Manglietia fordiana* var. *hainanensis* (Dandy) N. H. Xia; and the shrub of *Symplocos ovatilobata* Nooteboom, *Ardisia baotingensis* C. M. Hu, *Lasianthus chinensis* (Champion ex Bentham) Bentham, *Wendlandia uvariifolia* Hance, *Sterculia hainanensis* Merrill & Chun; and the herbs of *Hypolytrum nemorum* (Vahl) Sprengel, *Carex perakensis* C. B. Clarke, *Dianella ensifolia* (Linnaeus) Redouté, *Scleria terrestris* (Linnaeus) Fassett, *Ophiorrhiza cantonensis* Hance.

### Phenology

Flowering occurs from May and usually seeds maturity in June to July. Randomly collected 9 individual plants of the new species for morphological observations.

### Etymology

The epithen “*diaoluoshanica*” refers to the type locality in Diaoluoshan Mountain Natural Reserve.

### Additional Collections (paratypes)

CHINA. Hainan: Lingshui County, Diaoluo Shan Nature Reserve, moist place under forest, alt. 900 m, 21 June 2013, *Yang Hubiao 20130621* (four paratypes, TCGRI).

### Conservation status

The population of *Carex diaoluoshanica* comprises approximately 2 500 caespitose individuals, covers an area of 1 000 m^2^, at altitudes of 800–900 m. According to the IUCN (2001) category and criteria, *C. diaoluoshanica* is a vulnerable species(VU). Fortunately, this locality is in a remote place in the Diaoluoshan Natural Reserve.

### Relationships

*Carex* sect. *Lageniformes* consists of 12 species mainly distributed in eastern and southeast Asia, such as *C. breviscapa* C. B. Clarke (China, Indonesia, Japan, Malaysia, Myanmar, Philippines, Thailand, Vietnam, and Australia); *C. rhynchachaenium* C. B. Clarke (China, Philippines and Vietnam); *C. truncatigluma* C. B. Clarke (China, Malaysia, Philippines and Vietnam); *C. ascotreta* C. B. Clarke ex Franchet (China, Japan and Korea); *C. tenuispicula* Tang ex S. Yun Liang, *C. densipilosa* C. Z. Zheng & X. F. Jin, *C. ligata* Boott ex Bentham and *C. taihuensis* S. W. Su & S. M. Xu (all endemic to China); *C. yasuii* Katsuy (endemic to Japan); *C. palawanensis* Kük. (endemic to Philippines); *C. lageniformis* Nelmes and *C. pleurocaula* Nelmes (all endemic to Thailand). The new species is placed in *C*. sect. *Lageniformes* because its perigynium and nutlet are fusiform, and cylindric, slightly thickened and persistent style base (Fig. 1, H and J; Fig. 3, A and B). But according to observation its differ from these above-mentioned, the main difference of morphological characters beweeen *C. diaoluoshanica* and its related species see the identification key. In Hainan Island, two members of this section, *C. breviscapa* and *C. truncatigluma* were recorded previously. The new species can be easily distinguished from these by the petiolelike leaf base 5–10 cm long, and the subfiliform culm with only 2 spikes. *C. diaoluoshanica* is most closely related to *C. breviscapa* by characters of the perigynium: longer than the female glumes, fusiform, obscurely trigonous, minutely pubescent, many-veined, and with the base cuneate and stipitate, but it differs from by the subfiliform culms, wider leafblade with a petiolelike base and by having only 1 lateral female spike inserted near the culm base. In addition, the species *Carex longipetiolata* Q.L. Wang, H.B. Yang & Y.F. Deng is also recorded from Diaoluo Shan Nature Reserve[13]. This is similar to *C. diaoluoshanica* because it also has a petiolelike leaf base. However, *C. longipetiolata* belongs to *Carex* sect. *Rhomboidales* Kükenthal and is characterized by the involucral bracts surpassing the inflorescence; the trigonous, rhombic to ovoid perigynia with columniform bidentate apical beaks, and by the elliptic-rhomboid, trigonous nutlets that are constricted in the middle part. According to the above characteristics *C. longipetiolata* and *C. diaoluoshanica* are clearly different (Fig.1; Fig. 3, A–B and E–F).

*C. diaoluoshanica* can be distinguishing from its related species by the following key.

### Key to *Carex diaoluoshanica* and allies in section *Lageniformes* (Ohwi) Nelmes

1a Nutlets constricted at middle on angles.
  2a Culms, leaves, and bracts glabrous; leaves 2–4 mm wide..........*C. ascotreta*
  2b Culms, leaves, and bracts pilose; leaves 4–12 mm wide..........*C. densipilosa*
1b Nutlet not constricted on angles.
  3a Culms centra; lateral spikes female.
    4a Terminal spike androgynaeceous..........*C. palawanensis*
    4b Terminal spike male.
      5a Culms subfiliform, ca. 0.5 mm thick; leaves ensiform, base gradually narrowed into petiole-like structure..........*C. diaoluoshanica*
      5b Culms robust, 2–4 mm thick; leaves linear, base not petiole-like.
        6a Leaves as long as culms; perigynium glabrous..........*C. yasuii*
        6b Leaves far longer than culms; perigynium pubescent.
          7a Perigynium 3.5–5 mm; leaves 4–7 mm wide; culms 10–20 cm tall..........*C. breviscapa*
          7b Perigynium 5–6.5 mm; leaves 2–3 mm wide; culms 5–10 cm tall..........*C. rhynchachaenium*
  3b Culms axillary; lateral spikes female.
    8a Inflorescence equal to or longer than leaves.
      9a Male spikes linear, ca. 1 mm wide..........*C. ligata*
      9b Male spikes linear-cylindric, ca. 3 mm wide..........*C. taihuensis*
    8b Inflorescence shorter than leaves.
        10a Perigynium 2–3 mm..........*C. tenuispicula*
        10b Perigynium 3–6 mm.
          11a Inflorescence 0.7–1.2 cm long; lateral spikes 0.5–1 cm long; glume acute to obtuse at apex..........*C. lageniformis*
          11b Inflorescence 5–20 cm; lateral spikes 1.2–5 cm; glume usually with a short awn.
            12a All involucral bracts shorter than the extending spikelets..........*C. truncatigluma*
            12b Lower involucral bracts longer than the extending spikelets..........*C. pleurocaula*
